# National Insurance Bill—Second Reading

**Published:** 1911-06-03

**Authors:** 


### NATIONAL INSURANCE BILL—SECOND READING.

After a debate which lasted three days, the National Insurance Bill was read a second time in the House of Commons on May 29, and on the motion of the Chancellor of the Exchequer was committed to Committee of the whole House. The most remarkable feature of the debate was the unanimity with which all those speakers (and they included most of those who took part in the debate) who referred to the position of medical men under the proposed scheme, made it clear to the Chancellor of the Exchequer that before he could rely upon the whole-hearted support of the medical profession many alterations would have to be made in the provisions of the Bill. The promised statement by Mr. Lloyd George was not made until the third day; while it is apparent that he is anxious to propitiate the medical profession, his statement cannot be regarded as satisfactory; but, before making further reference to Mr. Lloyd George's statement, it will be interesting to summarise briefly those parts of the speeches of preceding speakers in which the provisions as to medical treatment were criticised. Mr. Forster pointed out that the whole of the great fabric which the Government proposed to erect depended upon the loyal and whole-hearted co-operation of the medical profession. So far as he could gauge the opinion of the medical profession at the present time, and it seemed to be expressed with extraordinary unanimity, it was that the provisions of the Bill as they stood would not do. He suggested that the doctors under the proposed scheme should be appointed by the local health committees. Mr. Barnes expressed the hope that the doctors would be treated generously. "If you have doctors poorly paid," he said, "only the poorest people will go to them, and there will be a social stigma attaching to those who do go."

Dr. J. Esmonde, who was listened to with attention, as men who speak from practical experience always are, said the Chancellor of the Exchequer had asked the medical profession to underwrite an insurance which he, with all his millions at his back, would not undertake. He explained to the House the origin of "club practice." "Many years ago it was possible for a medical man to employ unqualified assistants. He would give them thirty shillings a week and provide them with top hats and frock coats." But all that is completely altered now and it is no longer possible when an antiseptic dressing is required "for a medical man to put simply a rag round the finger of the patient." Proceeding, he said the profession thought there ought to be a free choice of medical men. Failing that, the club doctors of the present day would receive all the benefits of the Act, and those medical men who had not clubs at the present moment would see their patients, whom they had treated for years, and their families, perhaps, go over to other doctors. In conclusion, he expressed the opinion that if it was fair for one department of the Government—the Post Office—to pay 8s. 6d. for each healthy individual, he did not think another department could put the figures below that amount. If the Government was going to lower the standard of the profession, they would not have men of equal character coming into it to those who had come into it in the past, and he thought it would be to the advantage of the State to lift up the profession rather than to set it back.

Mr. Astor, in an interesting speech, strongly approved of the provisions embodied in the Bill for the erection of sanatoria and the treatment of consumption. Sir T. Whittaker, in referring to the fact that Friendly Societies had paid medical practitioners inadequately, said "Working men are the worst and hardest employers of others that you can have." Continuing, he said, "I do want this opportunity to be taken to put the remuneration of the doctors on a satisfactory footing. If you pay too little you will get the blacklegs, the failures, and the undesirables of the medical profession. . . The profession as a whole resent very strongly indeed the treatment to which they have been subjected in the past by Friendly Societies, and unless their position is to be very considerably improved there will be difficulty in working this scheme." Sir Thomas expressed the opinion that payment on a capitation basis was preferable to payment according to treatment, and he saw difficulties in the way of allowing patients to select their doctors. Sir Robert Finlay devoted the larger portion of his speech to the position of medical men under the proposed scheme. He said there was no profession in the country which gave more gratuitous labour for the benefit of the community than the medical profession. If any measure, however well intended, were passed which prejudicially affected the status and remuneration of the ordinary medical practitioner, it might have in the future a very serious and calamitous effect upon the profession as a whole. Sir Robert thought it most important that the patients should have the choice of their doctors; he also expressed the opinion that the remuneration that had been mentioned—six shillings, to include drugs, which would work out at four shillings or four shillings and sixpence for the doctor—was absolutely inadequate. He suggested that the control of the relations with the doctors should be in the hands of the health committees, and not in those of the Friendly Societies. In conclusion, he urged that the system must be such as would promote the self-respect both of the doctor and the patient; the ideal, in his view, was not a State doctor working by contract, but rather the relation which subsisted between a doctor called in by a patient who had confidence in him.

Mr. David Davies directed his comments mainly to the provisions dealing with the establishment of sanatoria, which he regarded as the most important in the Bill. He was prepared to admit that voluntary institutions could not deal with the problem of tuberculosis alone; the State must come to their assistance. He would like to see definitely established the principle that the grants from the State should be in some sort of proportion to the funds that are raised locally. Referring to the position of medical practitioners under the proposed scheme, he said nothing was more certain than that unless the medical profession was prepared to co-operate whole-heartedly the whole of the scheme would fall to the ground.

Dr. Addison, speaking as one who had been in close touch with medical organisation, said the medical profession was thoroughly desirous of co-operating in the measure in a hearty manner. He was confident that they would not desire for one moment that those who spoke on their behalf should put forward any ignoble considerations, or should pretend that class interests should supersede the general public interest. At the same time he felt certain that no party in the House would desire to treat the profession in any unfair spirit. He emphasised that one of the points on which the medical profession felt most strongly was the choice of a doctor, and he suggested that in any specified district all the medical men should claim the right to be enrolled upon a panel from which patients should be able to select their medical attendant. He thought it would be to the advantage of the societies if they were to allow the local health committees to manage the treatment of the sick. The first step which he thought must inevitably follow out of the Bill was that Poor Law infirmaries would have to come within its ambit, and he hoped the Chancellor of the Exchequer would allow the word " institution " to be used in a very literal sense. With regard to the question of fees, Dr. Addison expressed the opinion that in the case of people who joined the scheme voluntarily a higher capitation fee should be paid for higher incomes.

Mr. Gill thought it was a good idea to have a list of doctors among whom the patients would have a choice. Mr. Booth said his own estimate of what Friendly Societies would try to impose if the power was left with them was a capitation fee of 4s. per medical man, which he thought was niggardly. Mr. Bonar Law impressed on the Government that the whole of the medical profession was roused; he had received an enormous number of letters with regard to the position of doctors. Lord A. Thynne said that in the first place there must be free choice of doctors from a panel which should include all those who wished to serve upon that panel in any given district. As to the question of remuneration, the objection to the capitation system must increase if it were extended so as to include non-selected lives. Mr. Alden, who strongly supported the claims of the medical profession, expressed the opinion that the Bill would be an absolute failure without the support of the doctors; he advised the Chancellor to make a careful examination of the facts of the case. Mr. T. Taylor thought that if doctors asked they should be allowed to attend their own patients, and if patients asked to be attended by their own doctors their request should not be refused. Lord Charles Beresford expressed the hope that the doctors would be well looked after. Mr. Ramsay MacDonald said that not until the introduction of the Bill had there been any attempt to establish a system of social organisation which would use the doctor not merely for the purpose of attending to disease, but for eliminating it altogether. From the general point of view it was urgently necessary that medical men should have the fairest treatment. Mr. Austen Chamberlain asked what effect the Bill was going to have upon hospitals. His own view was that the Bill must inevitably lead to the assumption by the State of the cost of maintaining the hospitals of the country, an effect which he personally would deplore.

Mr. Lloyd George, in replying to the criticisms which had been made during the debate, dealt at length with the position of medical practitioners. His tone was conciliatory, but his statement was not altogether convincing. After recapitulating the main criticisms made on behalf of the medical profession he said they were all based on erroneous assumptions: payment would not necessarily be on a capitation basis, and the bad lives were all segregated to the Post Office contribution. He said that clause 14 did not put doctors under the heel of Friendly Societies; it did quite the reverse, for it provided that arrangements must be approved by the Insurance Commissioners, and they would act on the advice of the advisory committee, on which body medical men would be represented; so that in future, with doctors admitted to the health committees and the advisory bodies, the British Medical Association could make its terms with Friendly Societies. There was absolutely nothing in the Bill to prevent doctors entering into any of the arrangements they had been commending during the past week, and there was nothing to prevent them obtaining a large share of the twenty-five millions sterling which the State was raising. He had suggested payment at the rate of 6s. per head for medical advice and medicine because he had understood it was better than medical men expected, but the sum was neither fixed nor final. There was 6s. for a basis, a margin of half-a-crown, and provision for the ratepayer and taxpayer to come to the rescue if the charge grew. As to the free choice of doctors, he was in favour of a free choice within limits, but if they gave an unlimited free choice they might promote malingering. He believed the right course was to establish a panel of doctors, and while admitting that he regarded favourably the suggestion that the medical practitioners should be appointed by local committees, he made no promise that the provisions of the Bill, in this respect, would be altered. In conclusion he emphasised that the doctors, instead of being under the control of the Friendly Societies, would have an appeal to the Health Commissioners, and no contract would be entered into that was not satisfactory to them. Mr. Lloyd George spoke for two hours, and the debate was continued by a few other speakers until Mr. Asquith moved the closure, which was carried. The Bill was then read a second time. It is understood that the Government has not altered its intention of passing the measure this year.

